# Supplementary material for: Mitochondrial impairment and intracellular reactive oxygen species alter primary cilia morphology
Source: Life Sci Alliance. 2022 Sep 14;5(12):e202201505. doi: 10.26508/lsa.202201505 (PMC9475181; doi:10.26508/lsa.202201505)
Supplement: Supplementary file 1 [file LSA-2022-01505_TableS1.docx]

Table S1. List of the antibodies used for Western Blotting and immunofluorescence staining. Primary antibodies in bold.

| ANTIBODIES |  |  |  |  |
| --- | --- | --- | --- | --- |
|  | Species | Dilution | Company | Cat.N° |
| **Aquaporin2** | Mouse | 1:500 | Abcam | Ab15116 |
| **Na^+^K^+^ ATPase** | Mouse | 1:500 | Abcam | Ab76020 |
| **γ-tubulin** | Mouse | 1:5000 | Sigma | T6557 |
| **Acetylated α Tubulin** | Mouse | 1:1000 | Sigma | T6793 |
| **ARL13b** | Rabbit | 1:1000 | Proteintech | 17711-1-AP |
| **IFT88** | Rabbit | 1:1000 | Proteintech | 13967-1-AP |
| IRDye 680LT anti-mouse | Donkey | 1:10000 | Li-COR | 926-68022 |
| IRDye 680LT anti-rabbit | Donkey | 1:10000 | Li-COR | 926-32213 |
| Alexa 488 anti-mouse | Donkey | 1:1000 | Invitrogen | A21202 |
| Alexa 555 anti-rabbit | Donkey | 1:1000 | Invitrogen | A31572 |
| Anti-mouse IgG, HRP-linked | Horse | 1:1000 | Cell Signaling | 7076 |
