# Supplementary material for: Mitochondrial impairment and intracellular reactive oxygen species alter primary cilia morphology
Source: Life Sci Alliance. 2022 Sep 14;5(12):e202201505. doi: 10.26508/lsa.202201505 (PMC9475181; doi:10.26508/lsa.202201505)
Supplement: Supplementary file 2 [file LSA-2022-01505_TableS2.docx]

Table S2. List of the primers used for qPCR.

| Primers |  |  |
| --- | --- | --- |
|  | Forward | Reverse |
| **sod2** | TAACGCGCAGATCATGCAGCTG | AGGCTGAAGAGCGACCTGAGTT |
| **cat** | CGGCACATGAATGGCTATGGATC | AAGCCTTCCTGCCTCTCCAACA |
| **gpx1** | CGCTCTTTACCTTCCTGCGGAA | AGTTCCAGGCAATGTCGTTGCG |
| **gpx2** | GTGCTGATTGAGAATGTGGC | AGGATGCTCGTTCTGCCCA |
| **gpx3** | ATCCTGCCTTCTGTCCCTGCTC | TGGTGAGGGCTCCATACTCGTA |
| **gpx4** | CCTCTGCTGCAAGAGCCTCCC | CTTATCCAGGCAGACCATGTGC |
| **ucp2** | TAAAGGTCCGCTTCCAGGCTCA | ACGGGCAACATTGGGAGAAGTC |
| **hmbs** | CGGAGTCATGTCCGGTAAC | GGTGCCCACTCGAATCAC |
| **Tbp** | TGCTGTTGGTGATTGTTGGT | CTGGCTTGTGTGGGAAAGAT |
| **hprt** | CAGTCCCAGCGTCGTGATTA | GGCCTCCCATCTCCTTCATG |
